# Association between multiple serum biomarkers and cerebral vasospasm in patients with aneurysmal subarachnoid hemorrhage

**DOI:** 10.3389/fneur.2025.1592390

**Published:** 2025-06-05

**Authors:** Chenghao Yang, Fei Luo, Xi Chen, Yunliang Deng, Meirui Lan

**Affiliations:** Department of Neurosurgery, Zigong Fourth People's Hospital, Zigong, Sichuan, China

**Keywords:** predictive value, serum biomarkers, aneurysmal subarachnoid hemorrhage, cerebral vasospasm, machine learning

## Abstract

**Background:**

Cerebral vasospasm (CVS) is a potentially life-threatening complication for aneurysmal subarachnoid hemorrhage (aSAH) patients, raising their risk of disability and mortality substantially. Our study aimed to investigate whether serum biomarkers collected 24 h post-op can predict which aSAH patients would develop CVS within 14 days.

**Methods:**

We conducted a retrospective investigation of aSAH patients at our hospital during January 2020 to December 2023. For all patients, we collected comprehensive baseline information and multiple serum biomarkers. To analyze the correlation of these factors and CVS development, we applied univariate and multivariate logistic regression analysis and created a predictive model.

**Results:**

175 aSAH patients participated in our study – 84 of whom went on to develop CVS and 91 who did not. Four independent risk factors for CVS were determined through logistic regression analysis: Glasgow Coma Scale (GCS) score (OR = 4.633, 95% CI, 2.267–9.467, *p* < 0.001), hypoxia-inducible factor-1α (HIF-1α) (OR = 1.064, 95% CI, 1.025–1.104, *p* = 0.001), vascular endothelial growth factor (VEGF) (OR = 1.054, 95% CI, 1.033–1.076, *p* < 0.001), and endothelin-1 (ET-1) (OR = 1.151, 95% CI, 1.010–1.312, *p* = 0.035). When we incorporated these variables into the random forest and logistic regression predictive model, it performed exceptionally well, achieving an area under the receiver operating characteristic curve (AUC) of 0.966 and 0.959, respectively.

**Conclusion:**

Our findings suggest that GCS score and the biomarkers HIF-1α, VEGF, and ET-1 are independent predictors of CVS development in aSAH patients. Our predictive model based on the factors is remarkably accurate, offering clinicians a potentially valuable tool for early identification of high CVS risk patients.

## Introduction

Aneurysmal subarachnoid hemorrhage (aSAH) is a devastating type of stroke resulting from intracranial aneurysm rupture, with blood leaking into the extracerebral space and triggering a cascade of severe clinical presentations (1). This condition affects approximately 6.1 people per 100,000 globally each year, with an estimated 8.09 million cases globally—though these are extremely variable by region, age, and sex (2). The impact of aSAH extends beyond the initial event, most often leading to life-threatening complications like intracranial rebleeding, hydrocephalus, delayed cerebral ischemia, and cerebral vasospasm (CVS), and is a major public health concern worldwide (3).

Of these complications, CVS is particularly common and dangerous. CVS typically occurs between 3 and 14 days after the initial hemorrhage, with peak incidence on days 7 to 10 (4). CVS is defined by the narrowing of cerebral vessels, which reduces blood flow to brain tissue and causes ischemia and further neurological damage. Early diagnosis and intervention in CVS are vital to improving the outcomes of patients after aSAH (5).

While transcranial Doppler ultrasound is widely used to make CVS diagnoses, its accuracy is operator-dependent and does involve some level of subjectivity. Angiography, while absolute, is invasive and hence its application is limited. The modified Fisher grading system enables one to estimate the risk of CVS occurrence, but practitioners actually need some method of real-time, accurate diagnosis in every patient. This reality underlines the need for identifying objective, efficient, and non-invasive biomarkers of CVS prediction and diagnosis (6, 7).

Serum biomarkers are potential for CVS prediction in patients with aSAH because they can reflect the pathophysiological mechanisms of the condition, including inflammation, endothelial dysfunction, and vascular remodeling (8). For instance, hypoxia-inducible factor-1α (HIF-1α) is triggered by reduced oxygen and promotes a cascade of events that lead to blood vessel constriction and remodeling and could worsen CVS (9). Another is monocyte chemoattractant protein-1 (MCP-1), which promotes inflammation by activating immune cells to migrate to areas of injury, leading to damage to blood vessels and endothelium (10). Vascular endothelial growth factor (VEGF) may increase vascular permeability, cause endothelial dysfunction, and promote abnormal vascular remodeling. Its elevated level is one of the most important factors in the development of CVS and can predict well the occurrence of CVS after aSAH (11). Endothelin-1 (ET-1), being a potent vasoconstrictor, is increased after aSAH, causing sustained cerebral artery constriction, reduced blood flow, and promoting CVS. Researchers have noted peak plasma concentrations of ET-1 at 5 days post-hemorrhage to be highly correlated with the development of symptomatic vasospasm, making it a candidate as a screening biomarker (12).

However, their clinical application in individual biomarkers remains limited due to similar sensitivity and specificity. We aimed, in our study, to investigate the association between serum biomarkers MCP-1, HIF-1α, VEGF, and ET-1 and postoperative CVS occurrence in aSAH patients. We also aimed to determine whether machine learning models using these associated serum biomarkers and clinical variables could predict which aSAH patients would later develop CVS.

## Methods

### Study design and patients

Our study was a retrospective single-center study that was conducted after receiving approval from the Ethics Review Committee of the Fourth People’s Hospital of Zigong City. We adhered to the principles of the Helsinki Declaration, followed the Strengthening the Reporting of Observational Studies in Epidemiology (STROBE) guidelines, and complied with all applicable national regulations. Due to the retrospective collection of anonymized data, the ethics committee did not deem it necessary for patient consent to be obtained from the study participants.

We recruited patients with aSAH managed in our center between January 2020 and December 2023. For the purpose of having a well-defined study population, we used the following criteria:

Inclusion criteria:

1. Confirmation of aSAH by computed tomography (CT) upon hospital admission;
2. Aneurysm rupture verified through digital subtraction angiography (DSA);
3. Age of 18 years or older;
4. Initial diagnosis and hospital admission within 24 h of symptom onset;
5. Treatment with either craniotomy clipping or endovascular embolization surgery;
6. Availability of complete clinical and laboratory data.

Exclusion criteria:

1. Previous history of cerebrovascular disease or neurological disorders;
2. Development of serious complications during hospitalization (such as sepsis or liver and kidney dysfunction);
3. Presence of autoimmune or blood-related diseases;
4. Pregnancy or breastfeeding status;
5. Use of anticoagulant or antiplatelet medications within the previous 7 days;
6. Incomplete clinical data.

### Data collection

We gathered two primary categories of information from the de-identified patient records:

Clinical data: We collected and compared patient characteristics including age, gender, Glasgow Coma Scale (GCS) score, surgical approach, smoking history, presence of hypertension or diabetes, aneurysm location and diameter, cerebral hemorrhage volume, and Fisher grade.
Laboratory test data: From each patient, 5 mL of fasting venous blood was collected 24 h after surgery. We allowed the samples to stand for 30 min at room temperature, centrifuged them at 4°C and 1,000 × g for 15 min, and removed the upper serum for analysis. We measured serum MCP-1, HIF-1α, VEGF, and ET-1 concentrations by enzyme-linked immunosorbent assay (ELISA) using kits from Hangzhou Lianke Biotechnology Co., Ltd., Wuhan Feien Biotechnology Co., Ltd., and Shenggong Bioengineering (Shanghai) Co., Ltd.

The ELISA procedure included well preparation of blank wells and test sample wells, addition of 100 μL standard solution or diluted test sample (1:4 dilution with sample diluent), incubation at 37°C for 30 min, washing 5 times, addition of 50 μL of diluted antibody working solution, incubation, washing, addition of chromogenic reagent, and termination solution addition. We measured absorbance at 450 nm in an ELISA reader and calculated biomarker concentrations from standard curves.

As per international standards, CVS was characterized as middle cerebral artery velocity (VMCA) greater than 120 cm/s with severity grades of mild (120–140 cm/s), moderate (140–200 cm/s), and severe (>200 cm/s) (13). To assess CVS, we observed patients for 14 days after aSAH and performed daily or every other day transcranial Doppler (TCD) scans to measure cerebral blood flow velocity. Any occurrence of CVS, irrespective of severity, was considered as the outcome variable in our predictive model.

### Statistical analysis

We conducted all statistical analyses in Python 3.12. For continuous variables, we have reported means and standard deviations, and for categorical variables, counts and percentages. We have employed independent *t*-tests or χ^2^ tests for univariate clinical data analysis and reported results as odds ratios (ORs) with 95% confidence intervals (CIs). Variance Inflation Factor (VIF) is used to test multicollinearity of variables, and value of 5 or below for all variables is not comparable. Missing data were handled by Multiple Imputation by Chained Equations (MICE, 20 imputations) with R-hat <1.1 being a sign of convergence; Rubin’s rules averaged results with categorical variables imputed by mode and one-hot encoded, and continuous variables imputed by median and standardized.

All the variables were first assessed using univariate logistic regression, and those with statistical significance or clinical relevance were included in a multivariate model. Stepwise selection procedure was used to determine independent predictors. The findings were presented as coefficients, *p*-values, odds ratios (ORs), and 95% confidence intervals (CIs). The data were split randomly into training and validation sets in a 7:3 ratio for model development and evaluation. Independent risk factors were used as input variables to develop logistic regression and random forest models. The logistic regression model employed L2 regularization with a maximum of 1,000 iterations to prevent overfitting. The random forest model was limited to a depth of 4 at most with 100 trees for controlling complexity. For avoiding overfitting of the random forest model, 5-fold cross-validation was applied, and grid search was applied for hyperparameter tuning. Receiver operating characteristic (ROC) curve parameters, area under the curve (AUC), sensitivity, and specificity were determined for both models to assess their predictive accuracy in determining postoperative CVS in aSAH patients as well as to identify the optimal model. *p* < 0.05 was considered to be the level of statistical significance.

## Results

### Characteristics of participant

**Figure 1:**
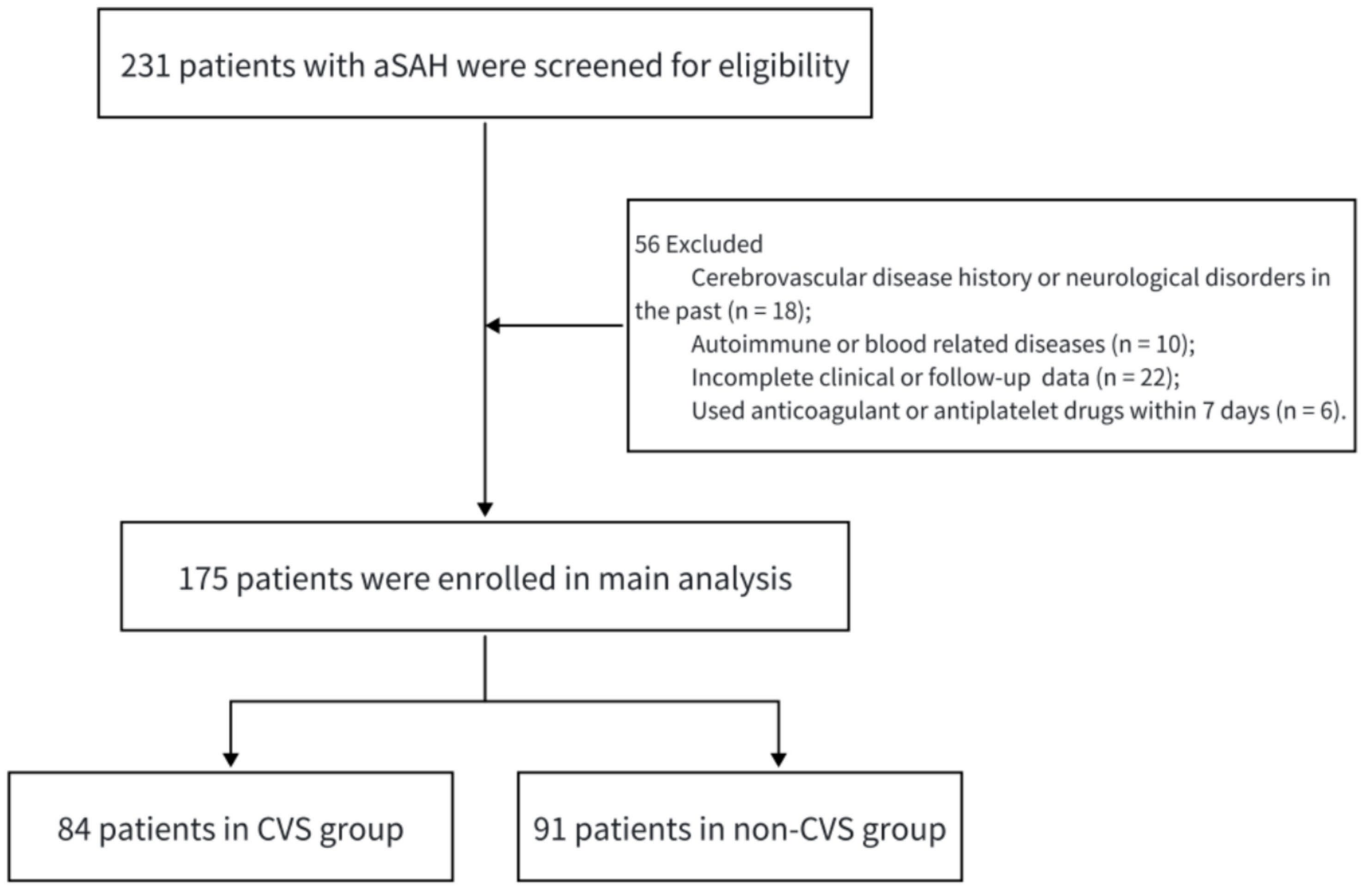
Flow diagram depicting the participant selection process.

175 patients with aSAH were studied by us, out of which 84 developed CVS and 91 did not. Figure 1 illustrated our participant recruitment. There was no heterogeneity other than some clinical parameters. The CVS group had significantly increased intracerebral hemorrhage volumes (20.52 ± 4.51 vs. 18.75 ± 2.75) and decreased GCS scores (13.04 ± 1.83 vs. 13.78 ± 1.10). Also, a greater proportion of CVS patients had aneurysms ≥10 mm in diameter at 27.38% compared to 19.78% among non-CVS patients. The average time from postoperative sample collection to onset of CVS was 5.48 days (Table 1).

**Table 1:** Characteristics of patients in the CVS and non-CVS group at baseline.

Variable	CVS group (*n* = 84)	Non-CVS group (*n* = 91)	*p*-value
Age (year)	60.63 ± 8.82	61.41 ± 10.23	0.59
Gender			0.27
Male	40 (47.62%)	52 (57.14%)	
Female	44 (52.38%)	39 (42.86%)	
Diabetes history			0.13
Yes	15 (17.86%)	25 (27.56%)	
No	69 (82.14%)	66 (72.44%)	
Hypertension history			0.02
Yes	76 (90.48%)	70 (76.92%)	
No	8 (9.85%)	21 (23.08%)	
Smoking history			0.72
Yes	28 (33.33%)	28 (30.59%)	
No	56 (66.67%)	63 (69.41%)	
Aneurysm location			0.58
Carotid artery system	52 (61.90%)	61 (67.03%)	
Vertebrobasilar artery system	32 (38.10%)	30 (32.97%)	
Aneurysm diameter	8.43 ± 1.58	8.29 ± 1.54	0.54
Intracerebral hemorrhage volume	20.53 ± 4.52	18.75 ± 2.75	<0.01
GCS score	13.04 ± 1.83	13.78 ± 1.10	<0.01
Fisher grade			0.07
2	28 (33.33%)	36 (39.56%)	
3	44 (52.38%)	51 (56.04%)	
4	12 (14.29%)	4 (7.27%)	
Treatment methods			0.87
Endovascular embolization	76 (90.47%)	84 (92.31%)	
Craniotomy clipping	8 (9.53%)	7 (7.69%)	
Postoperative CVS onset time	5.48 ± 2.62	–	–

CVS, cerebral vasospasm; GCS, Glasgow Coma Scale.

### Comparison of multiple serum biomarkers levels in patients with aSAH

**Figure 2:**
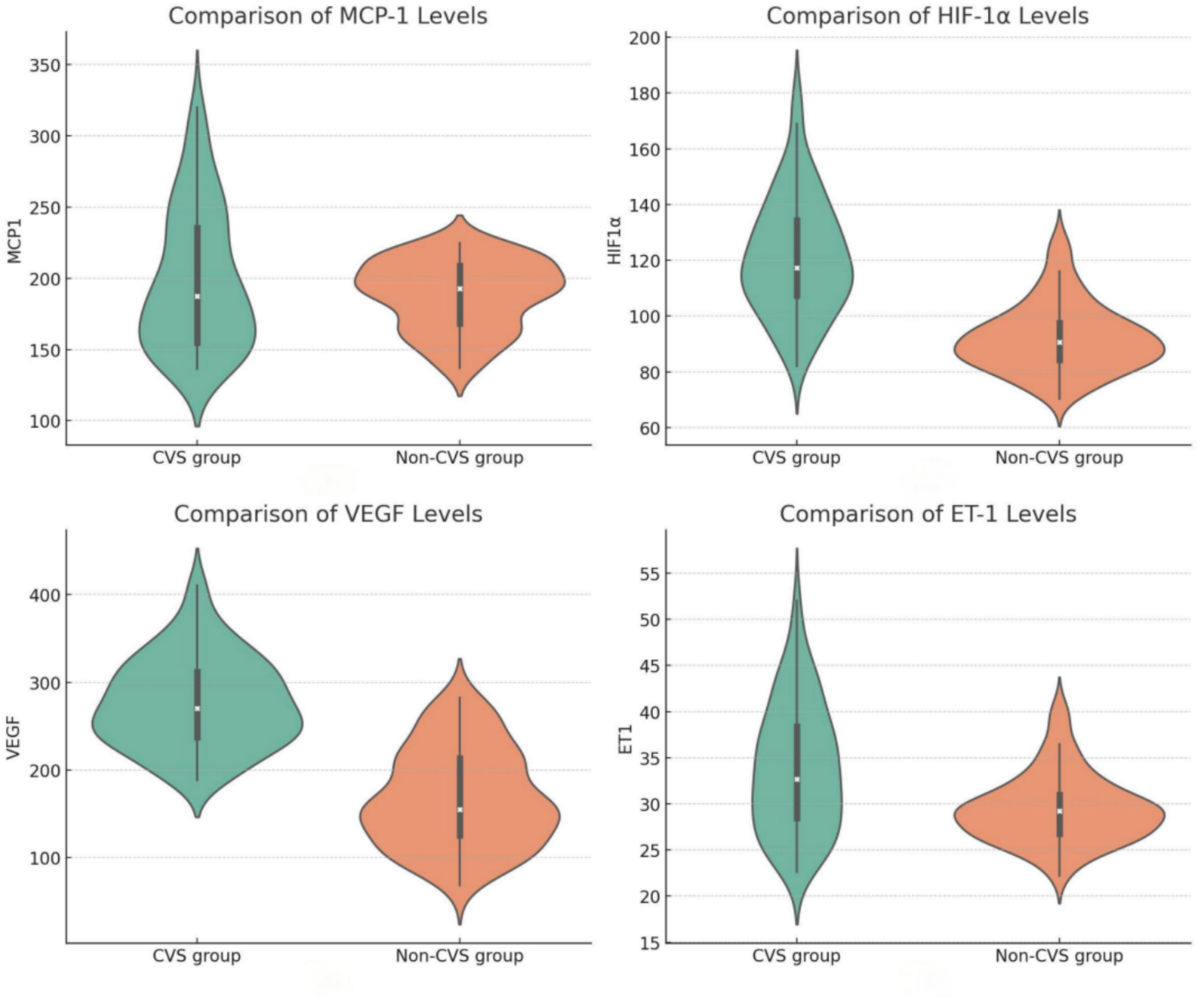
Comparison of serum MCP-1, HIF-1α, VEGF, and ET-1 levels in patients with aSAH.

As indicated in Table 2, we found striking differences between the CVS and non-CVS group in serum biomarker concentrations. Individuals with CVS contained far higher amounts of HIF-1α at 121.33 ± 20.73 pg./mL compared with a mere 92.09 ± 12.14 pg./mL for the non-CVS population (*p* < 0.001). For VEGF, it was even higher among CVS, where they stood at 276.84 ± 51.32 pg./mL while in the non-CVS it was at 167.61 ± 49.89 pg./mL (*p* < 0.001). ET-1 was also markedly elevated in the CVS group (33.78 ± 6.94 pg./mL vs. 29.53 ± 3.62 pg./mL, *p* < 0.001). MCP-1 was not significantly different between groups (*p* = 0.081) (Figure 2).

**Table 2:** Comparison of serum MCP-1, HIF-1α, VEGF, and ET-1 levels between two groups of patients.

Biomarkers	CVS group	Non-CVS group	*t*	*p*
MCP-1 (pg/ml)	198.64 ± 48.58	188.57 ± 24.22	1.75	0.081
HIF-1α (pg/ml)	121.33 ± 20.73	92.10 ± 12.14	11.49	<0.001
VEGF (pg/ml)	276.84 ± 51.32	167.61 ± 54.90	13.57	<0.001
ET-1 (pg/ml)	33.83 ± 6.87	29.45 ± 3.71	5.30	<0.001

CVS, cerebral vasospasm; MCP-1, monocyte chemoattractant protein-1; HIF-1α, hypoxia-inducible factor-1α; VEGF, vascular endothelial growth factor; ET-1, endothelin-1.

### Univariate analysis of CVS in patients with aSAH

Our univariate analysis revealed several factors that were significantly associated with the development of CVS. These included increased intracerebral hemorrhage volume (*p* = 0.002), decreased GCS score (*p* = 0.002), and previous history of hypertension (*p* = 0.019). Of biomarkers, HIF-1α, VEGF, and ET-1 were strongly associated with CVS development (*p* < 0.001) (Table 3).

**Table 3:** Univariate analyses of cerebral vasospasm in patients with aneurysmal subarachnoid hemorrhage.

Variable	Coefficient	t/x^2^	*p* value	OR	95% CI
Age	−0.008	−0.538	0.591	0.992	0.961–1.023
Male	−0.383	−1.295	0.208	0.682	0.376–1.238
Intracerebral hemorrhage volume	0.129	3.025	0.002	1.138	1.046–1.237
Aneurysm diameter	0.061	0.609	0.543	1.061	0.876–1.286
GCS score	−0.334	−3.108	0.002	0.716	0.581–0.884
Diabetes	−0.555	−1.504	0.133	0.574	0.278–1.183
Hypertension	1.047	2.342	0.019	2.849	1.186–6.848
Smoking	0.118	0.363	0.716	1.125	0.596–2.124
Vertebrobasilar artery	0.224	0.708	0.479	1.251	0.673–2.327
Fisher grade	0.427	1.712	0.087	1.533	0.941–2.498
Endovascular Embolization	−0.234	−0.432	0.666	0.792	0.274–2.287
MCP-1	0.007	1.729	0.084	1.007	0.999–1.015
HIF-1α	0.112	6.784	<0.001	1.119	1.083–1.156
VEGF	0.039	6.639	<0.001	1.039	1.028–1.051
ET-1	0.151	4.569	<0.001	1.163	1.090–1.241

GCS, Glasgow Coma Scale; MCP-1, monocyte chemoattractant protein-1; HIF-1α, hypoxia-inducible factor-1α; VEGF, vascular endothelial growth factor; ET-1, endothelin-1.

### Multivariate logistic regression analyses of CVS in patients with aSAH

To find independent predictors of CVS, we performed multivariate logistic regression analysis (Table 4). We found that four parameters were independently related to the development of CVS: GCS score (OR = 4.633, 95% CI, 2.267–9.467, *p* < 0.001), HIF-1α (OR = 1.064, 95% CI, 1.025–1.104, *p* = 0.001), VEGF (OR = 1.054, 95% CI, 1.033–1.076, *p* < 0.001), and ET-1 (OR = 1.151, 95% CI, 1.010–1.312, *p* = 0.035). The VIFs were all under the value of 5.

**Table 4:** Multivariate logistic regression analyses of cerebral vasospasm in patients with aneurysmal subarachnoid hemorrhage.

Variable	Coefficient	SE	Wald χ^2^ value	*p* value	OR	95% CI	VIF
GCS score	1.533	0.375	14.844	<0.001	4.633	2.267–9.467	1.873
HIF-1α	0.062	0.019	10.861	0.001	1.064	1.025–1.104	2.092
VEGF	0.053	0.011	24.852	<0.001	1.054	1.033–1.076	2.470
ET-1	0.141	0.067	4.972	0.035	1.151	1.010–1.312	1.739

GCS, Glasgow Coma Scale; HIF-1α, hypoxia-inducible factor-1α; VEGF, vascular endothelial growth factor; ET-1, endothelin-1; VIF, variance inflation factor.

### Prediction model validation

**Figure 3:**
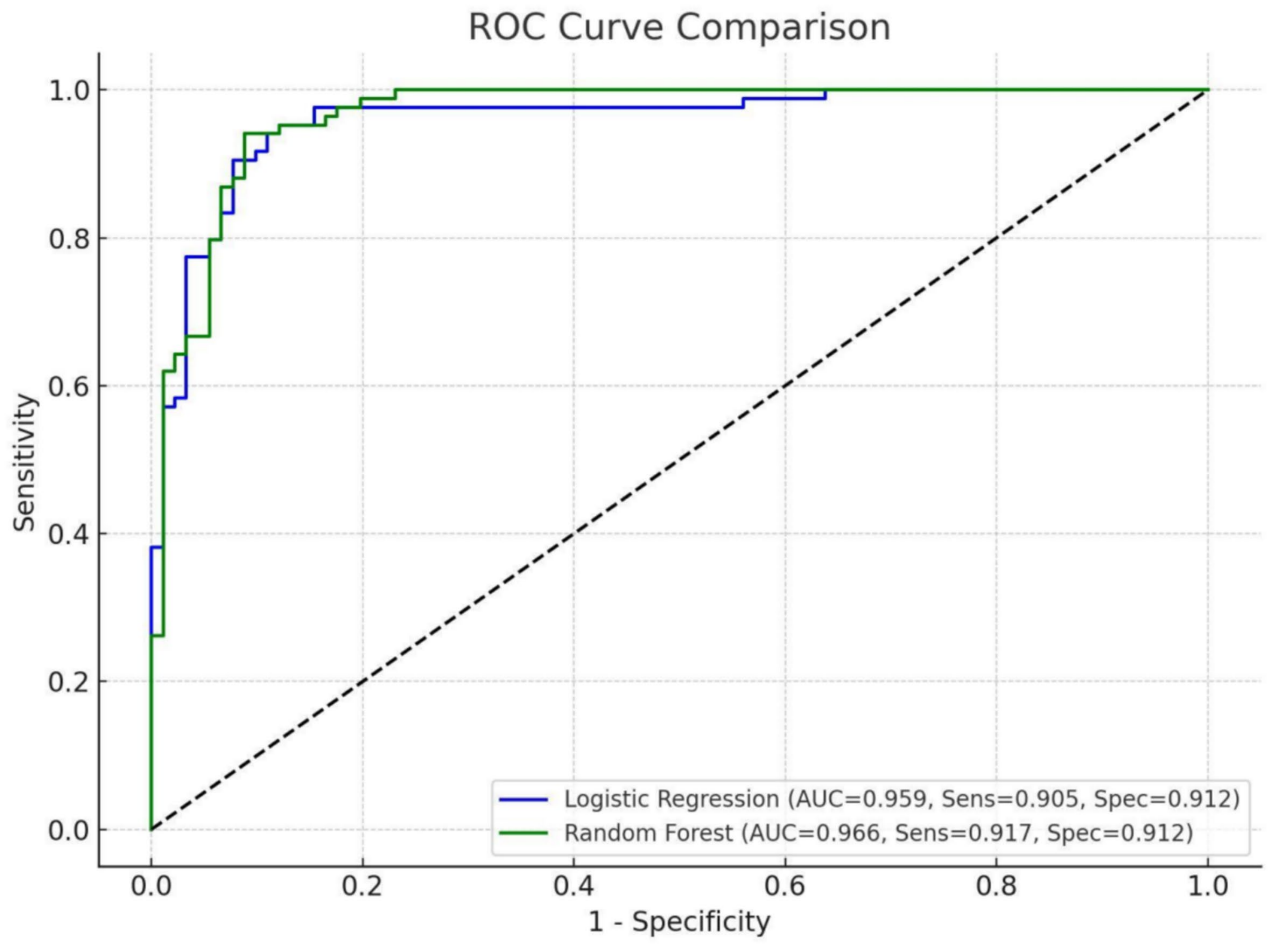
ROC curve for combined prediction model.

After we identified these independent risk factors, we combined them to create a model for the prediction of CVS development. The random forest model achieved an AUC of 0.966 with a sensitivity of 91.7% and specificity of 91.2%, while the logistic regression model yielded an AUC of 0.955, with a sensitivity of 90.5% and the same specificity of 91.2% (Figure 3).

## Discussion

We examined, in our study, the relationship between some of the serum biomarkers and development of CVS in patients with aSAH. In logistic regression in our study, we established that GCS score and three serum biomarkers, HIF-1α, VEGF, and ET-1, were all statistically related to occurrence of CVS. Even though MCP-1 was related to CVS, the relation did not prove to be statistical. Of particular interest was our random forest model combining GCS score with these biomarkers, which possessed great predictive potential, with ROC curve analysis identifying its potential to identify at-risk patients early on.

Our findings are in agreement with several previous studies of serum biomarkers for the prediction of CVS. Researchers discovered that levels of HIF-1α, ET-1, and matrix metalloproteinase-9 (MMP-9) were markedly elevated in individuals who developed CVS following aSAH, and the levels of biomarkers were positively associated with CVS severity (14). Similarly, a composite model comprising serum macrophage migration inhibitory factor (MIF), C-reactive protein (CRP), and interleukin-6 (IL-6) also showed good AUC for predicting aSAH patient outcome (15). Another study found that serum levels of VEGF, MMP-9, and von Willebrand factor (vWF) were significantly greater in aSAH patients who subsequently developed CVS compared to those who did not, with these biomarkers increasing several days before CVS development (16). Most recently, a multi-center United States study utilized the LightGBM model to compare various blood and cerebrospinal fluid biomarkers paired with clinical information to predict CVS risk with AUC values above 0.80 at various time points (17).

Furthermore, Zhao et al. showed that ET-1 was strongly predictive of prognosis with an AUC of 0.948 (18). Although the measurement time points differed from ours, the similar AUC indicates that, despite ET-1 levels typically peaking 3 to 7 days after subarachnoid hemorrhage, ischemic and inflammatory responses have already begun to emerge within 24 h post-surgery, potentially reflecting early pathophysiological changes (19, 20). However, we found some discrepancies in our findings with other studies regarding the predictive performance of certain biomarkers. Although Pu et al. noted that serum MCP-1 was found to have good predictive value for CVS with much higher concentrations in the post-operative CVS group, no significant correlation between MCP-1 and CVS was noted in our study (21). This paradox highlights the multifactorial etiology of CVS pathophysiology, with a range of factors including altered vascular smooth muscle function and endothelial damage, with different inflammatory factors potentially causing CVS formation in different ways (22). In addition, the effect of serum MCP-1 could be modified by the stage of the immune response, subject variability, and different vascular wall and endothelial conditions—albeit possibly causing no considerable correlation in our research (23).

Although past biomarkers and clinical variables had made significant advancements in CVS prediction, the traditional models never fully considered individual patients’ differences and the complexity of clinical characteristics and therefore lost the capability to fully represent the pathophysiological states of the patients (24–26). In recent years, the application of machine learning methods in medicine has offered a new way to predict diseases. Through the incorporation of various biomarkers and clinical data, machine learning models could integrate data from multiple sources and made more accurate, specific, and sensitive predictions (27–29). More specifically in more accurately fitting individual variability in aSAH patients, it rendered it a more comprehensive, non-invasive means of predicting aSAH patient prognosis (30, 31). Accordingly, future research will have to include advanced machine learning techniques capable of processing multiform types of data like other clinical variables including imaging data and gene expression in order to allow better representation of heterogeneity among patients. We are aware of a couple of limitations in the current study. For one, its retrospective nature has the potential of introducing biases and the small sample size relative to the number of the variables poses a threat of model overfitting. External prospective validation through follow-up studies will be required for this to be resolved. Second, the current study is a single-center study with a fairly small population, and multi-center validation studies should be carried out to examine the generalizability of the prediction model in more heterogeneous populations and various medical settings. Third, despite having assessed several biomarkers, patient-to-patient variation in biomarker levels and the potential influence of unmeasured confounders could have influenced our predictive models’ accuracy and reliability. Last, by assaying biomarkers at a single point in time (24 h post-surgery), we may not have best represented their dynamic characteristics in aSAH and CVS.

## Conclusion

Our research indicates the extensive promise of serum biomarkers, in particular, HIF-1α, VEGF, and ET-1, with machine learning models in predicting CVS following aneurysmal subarachnoid hemorrhage. Nevertheless, to improve such models and to advance clinical practice, additional research involving larger patient groups and serial measurement of biomarkers is needed.

## Data Availability

The original contributions presented in the study are included in the article/supplementary material, further inquiries can be directed to the corresponding author.
